# Cross-feedback with Partner Contributes to Performance Accuracy in Finger-tapping Rhythm Synchronization between One Leader and Two Followers

**DOI:** 10.1038/s41598-019-43352-x

**Published:** 2019-05-24

**Authors:** Taiki Ogata, Takahiro Katayama, Jun Ota

**Affiliations:** 1 Research into Artifacts, Center for Engineering (RACE), The University of Tokyo, Kashiwano-ha, 5-1-5, Kashiwa Chiba, 277-8568 Japan; 2 Department of Precision Engineering, School of Engineering, The University of Tokyo, Hongo 7-3-1, Bunkyo, Tokyo, 113-8656 Japan

**Keywords:** Psychophysics, Human behaviour

## Abstract

As observed in musical ensembles, people synchronize with a leader together with other people. This study aimed to investigate whether interdependency with a partner improves performance accuracy in rhythm synchronization with the leader. Participants performed a synchronization task via auditory signal by finger tapping in which two followers simultaneously synchronized with a leader: an isochronous metronome or a human leader with or without feedback from the followers. This task was conducted with and without cross-feedback (CFB) between the followers. The followers’ weak mutual tempo tracking via the CFB and the followers’ strong tempo tracking to the leader improved the tempo stability. Additionally, because the interdependency between the followers was weaker than the followers’ dependency on the human leader, the CFB did not enlarge the synchronization error between the human leader and the followers, which occurred in synchronization with the metronome. Thus, the CFB between the followers contributed to accuracy in synchronization with the human leader. The results suggest that in ensembles, players should strongly attend to the leader and should attempt to be less conscious of partners to maintain the appropriate balance between influences from the leader and partners.

## Introduction

As seen in the arts of music and dance, human beings naturally synchronize with each other’s movement. This temporal coordination of motor rhythm with external rhythm is characteristic of human behaviour^1^. People can correct the timing of their own movements during synchronization on a millisecond time scale within a wide range of tempos^1,2^. In addition, people synchronize with external rhythms simultaneously with other people. For example, a player synchronizes with a conductor in an orchestra together with other players, and people dance with partners when synchronizing with music. Does feedback from a partner improve performance accuracy in rhythm synchronization to external signals, or does it work as a distractor for rhythm synchronization? If performance is improved by a partner’s feedback, what temporal dependency on the partner causes the improvement?

Mates *et al*.^3^ investigated the effect of a participant’s partner’s feedback on the participant’s synchronization with external rhythm in the literature on social feedback^4^, which involves cross-linking between the sensory and motor systems of two individuals. The authors conducted an auditory synchronization task in which a participant tapped his or her fingers in an isochronous sequence together with and without a partner who synchronized the same sequence simultaneously. The asynchrony between the timings of the sequence and the participant’s tapping increased as a result of the cross-feedback (CFB) condition in which the participant perceived the partner’s feedback compared to synchronization without the CFB. The authors did not find mutual dependency on the synchronization performance with the partner’s feedback. These results suggest that the CFB with a partner is only a distractor in synchronization with the external rhythmic sequence. In fact, the timing of rhythm production with the external sequence is influenced by distractor stimuli presented together with the sequence^5–7^.

One might consider that if highly trained musicians perform the same task, they can show high accuracy and some mutual dependence with other players. In fact, asynchrony and its variability are lower for trained musicians than for untrained people^8–10^. For example, drummers showed smaller asynchrony and lower variability of tempo in synchronization with auditory signals by finger tapping than did non-musicians^11^. In addition, some studies have found temporal dependencies between players in a musical ensemble of professional musicians^12–14^. For example, Wing *et al*. and Timmers *et al*. investigated the rhythm synchronization between four participants^13,14^. In a string quartet, the players adjusted their own timing using asynchrony between their own and other players’ timing^13^. Additionally, there were some temporal mutual dependencies between the players, such as mutual tracking of tempo^13,14^. The intense, long-term training of musicians influences the performance accuracy and temporal dependencies they show when playing an instrument with other players. However, these temporal features in rhythm synchronization may have been found in studies because the participants were highly trained musicians.

There is another possible reason why low performance and no mutual dependency were found in previous research^3^: The participants were asked to synchronize with a constant-tempo metronome. Recently, many studies have found unique characteristics of dyad rhythm synchronization that differ from isochronous sequences^15–22^. Konvalinka *et al*. conducted a synchronization task in which participants attempted to maintain a target tempo with auditory feedback from their partner, who simultaneously maintained the same tempo^18^. The synchronization errors (SEs) of tapping between the participants and the partners were not worse than those with the constant-tempo metronome. In addition, the authors found mutual tracking of inter-tap intervals (ITIs) between the participants under the CFB condition where the two participants obtained feedback on their partner’s tapping. That is, if each follower’s tempo sped up at the last tap, the other tempo sped up, and if each follower’s tempo slowed down, the other slowed down. Thus, the participants did not create a leader-follower relationship but became followers of their partner at the same time. The authors called this mutual dependency “hyper-followers”. To investigate the characteristics of ITI drift in the pair context, Okano *et al*. used the synchronization-continuation (SC) task^22^. In the SC task^23–25^, the participants were first asked to synchronize with an isochronous sequence and then to keep the same tempo after the metronome had stopped. Okano *et al*. conducted this SC task with and without a partner and found that the ITI gradually decreased in the SC task with a partner.

These previous studies show differences in performance accuracy and temporal dependency with dyad rhythm synchronization from isochronous sequences. Thus, the synchronization between a human leader and a follower should also be different from that with a metronome. For example, the interaction between a human leader and followers would create coupling of their timing, and the SEs would not increase. In addition, the performance of rhythm synchronization with external stimuli has been investigated by indexes other than SEs^1,2^, such as tempo-keeping accuracy, tempo stability, and variability of SEs. In particular, the CFB of the followers would increase their tempo stability. The mutual dependency of tempos^18^ is believed to occur unconsciously in dyad synchronization. Therefore, followers who synchronize with a human leader would depend on each other in the same manner by the CFB between them even though they intend to synchronize with the leader. The mutual dependency of their tempos could help them decrease their tempo variability. The purpose of this study was to investigate the temporal accuracies of people in rhythm synchronization with a human leader together with and without a partner who attempt to synchronize with the leader at the same time. Accuracy was investigated from various perspectives, i.e., tempo-keeping accuracy, tempo stability, SEs, and the variability of SEs. In addition, this study aims to reveal what temporal dependencies between the leader and the follower and between followers affect performance accuracy.

**Figure 1:**
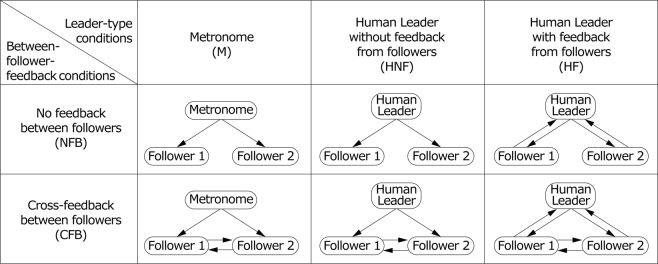
Conditions used in the present experiment. The arrows indicate the flows of timing information. For example, the arrow from the human leader to follower 1 means that follower 1 perceived the tap timing of the human leader via auditory signals. Three leader types and two between-follower-FB conditions were prepared. Under the M conditions, the followers synchronized to a constant tempo (700 ms) metronome. Under the HNF and HF conditions, the followers synchronized to a human leader who was asked to keep a constant tempo (700 ms) without or with feedback on tap timing from the followers, respectively. In the HNF condition, the human leaders tapped their index fingers to keep the tempo without any external stimuli after the 700 ms tempo pacemaker had been stopped. That is, they performed a continuous finger-tapping task. Under the NFB condition, the followers synchronized to the leader separately, and under the CFB condition, the followers perceived their partner’s tap timing.

For these purposes, we conducted an auditory synchronization task using finger tapping between one leader and two followers. The followers were required to synchronize with a constant-tempo metronome or a human leader who was asked to keep a tempo. This task was performed under three leader-type conditions and two between-follower conditions (Fig. 1). The leaders were a constant tempo (700 ms) metronome (M condition) and a human leader who attempted to maintain a target tempo (700 ms) without or with feedback from the followers (HNF and HF conditions, respectively). Under these leader conditions, the followers synchronized with the leader’s timing presented via auditory signals without or with feedback from their partner (NFB and CFB conditions, respectively). At the beginning of the trials, an auditory constant-tempo (700 ms) pacemaker was presented to the followers in all conditions and to the human leader in the HNF and HF conditions. After the pacemaker stopped, the participants started the finger-tapping tasks. The leader’s tap timing was presented to the followers as a 500 Hz pure tone, and the followers’ tap timing was submitted to the leader and the partner as 1,000 or 2,000 Hz, respectively.

## Results

Eighteen participants (6 groups) joined the present experiment. We used the data from all groups for the analyses. Due to space limitations, we show part of the results of the statistical tests in this section. All results are shown in the Supplemental Information section. Because we did not find any significant differences in the pitch of auditory stimuli at which the followers’ responses were presented (1,000 or 2,000 Hz), the results of two followers are integrated below.

### Accuracy and variability of followers’ tapping performance

**Figure 2:**
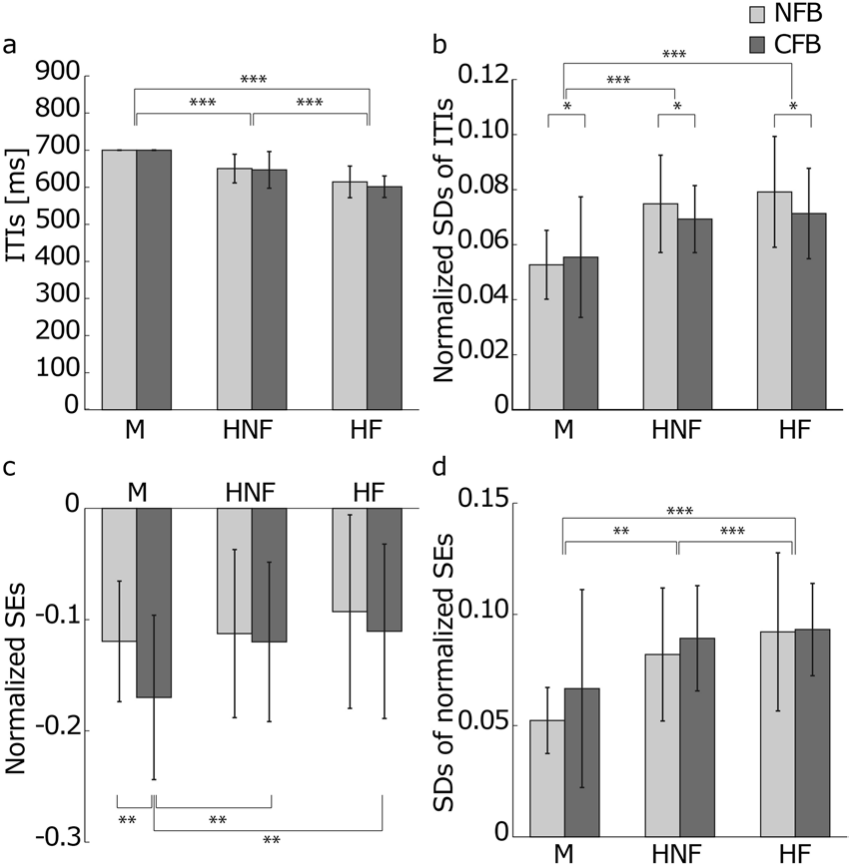
The temporal accuracy and variability of the followers’ tapping. (**a**) Means of followers’ ITIs under each condition. This index depicts the accuracy of followers’ tempo keeping. The CFB between the followers did not affect the followers’ tempos. Under the human leader conditions, the tempo of the followers increased compared to the metronome condition, and the feedback from the followers to the leader further accelerated the tempo. (**b**) The normalized SDs of the followers’ ITIs. This graph shows followers’ tempo variability. When the value is 0.1, the SDs of the follower’s tempo is 10% of the averaged leader’s ITIs in each condition. The CFB between the followers decreases tempo variability. (**c**) Means of normalized SEs between the leader and the followers. This index shows the followers’ synchronization accuracy. When the value is 0.1, the asynchrony between the taps of the leader and the follower is 10% of the corresponding leader’s ITI. The negative values indicate that the followers’ finger taps attended to the leader’s taps. The CFB between the followers increased the synchronization error under the metronome condition but not under the human leader conditions. (**d**) The average SDs of the normalized SEs between the leader and the followers. This value indicates the variability of the asynchrony between the leader and the followers. A value of 0.1 means that the SD of the SEs is 10% of the corresponding leader’s ITIs. The CFB between the followers did not affect the variability of SEs. The error bars indicate the SDs between the trials. *, ** and *** represent *p* < 0.05, *p* < 0.01 and *p* < 0.001, respectively.

First, to investigate the accuracy and stability of the participants’ tapping performance, we calculated four indexes: the mean and the standard deviations (SDs) of followers’ ITIs and those of the SEs between the leader and the followers. The ITIs comprise an index of tempo-keeping accuracy. The closer the ITI is to the target tempo, 700 ms, the better the participants keep the tempo. The smaller the SDs of ITIs, the smaller the tempo variability is. The SEs between the leader and the follower are asynchronies of corresponding tap onsets between them. The SE between the leader and the follower below is referred to as the SE. The closer the SE is to 0, the better the followers synchronize to the leader. The smaller the SDs of SEs, the more followers tap before or after a constant period from the corresponding leader’s taps. The mean and variability of SEs and the variability of ITIs are affected by the tempo of the external stimuli. The SEs increase with an increase in the inter-onset intervals (IOIs) of external sequences^26–29^. The variability of SEs and ITIs also increases with the increase in IOIs^30–34^. In addition, the tempo gradually increases or decreases in continuous tasks in which people attempt to keep a target tempo^24,25^. These findings suggest that if the tempo changes during each trial in the present experiment, the mean and the variability of SEs and the variability of ITIs cannot be directly compared. Therefore, we normalized the SEs using the leader’s ITIs corresponding to each SE. In addition, the SDs of ITIs were divided by the means of the leader’s ITIs in each trial. Figure 2 shows the results.

There was no effect of the CFB between the followers on the means of the followers’ ITIs (Fig. 2a). On the other hand, the means of the followers’ ITIs were smaller under the HNF conditions than under the M conditions and were the smallest in the HF conditions. A three-way mixed-design ANOVA with the pitch, leader type, and between-follower FB revealed no significant difference in the between-follower-FB conditions (*p* = 0.072). We found a significant difference in leader type (*p* < 0.001). Multiple comparisons showed that the mean of the ITIs was significantly smaller under the M conditions than under the HNF and HF conditions, and it was significantly smaller under the HNF conditions than under the HF conditions (all *p* < 0.001). These results showed that the CFB between followers did not affect followers’ tempos; additionally, follower’s tempo were faster under the human leader conditions than under the M conditions, and the feedback from the followers to the leader sped up the followers’ tempos.

The CFB between the followers decreased the normalized SDs of ITIs (Fig. 2b). The tempo variabilities were higher under the HNF and HF conditions than under the M conditions. A three-way mixed-design ANOVA showed that the normalized SD of ITIs was significantly smaller under the CFB conditions than under the NFB conditions (*p* = 0.031). In addition, we found a significant difference in leader type (*p* < 0.001). Multiple comparisons revealed no significant differences between the HNF and NF conditions (*p* = 0.145). The normalized SD of the ITIs under the M conditions was significantly smaller than that under the HNF and HF conditions (*p* < 0.001 and *p* < 0.001, respectively). Thus, the variability of the followers’ tempo decreased because of the CFB between the followers. In the synchronization with the human leader with and without the feedback from followers, the variability of the follower’s tempo was higher than it was in the synchronization with the metronome.

Under all conditions, the means of the normalized SEs (Fig. 2c) were negative. The negative value of the SEs means that the followers tended to tap before the timing of the metronome and the taps of the human leader. People usually show *negative mean asynchronies* (NMAs) during synchronization with a metronome using finger tapping^1,2,10,35,36^. The results of our experiment show that NMAs are also observed during synchronization with human leaders regardless of whether a partner is present. Only the NMA was larger under the M-CFB condition than under the other conditions. Comparing the SEs between the conditions, we conducted a three-way mixed-design ANOVA in which the factors were pitch, leader type, and between-follower FB. The result showed a significant interaction between leader type and between-follower FB (*p* = 0.016). A *post hoc* test revealed that under the M condition, the normalized SEs were significantly larger under the CFB condition than under the NFB condition (*p* = 0.001). As was the case in a previous study^3^, the SEs were larger under the M-CFB condition than under the M-NFB condition. However, we found no significant differences between the NFB and CFB conditions under the HNF or HF conditions (*p* = 0.638 and *p* = 0.055, respectively). In addition, we found a significant difference between leader type under the CFB condition (*p* = 0.001). Multiple comparisons revealed that the normalized SEs were significantly higher under the CFB condition than under the HNF and HF conditions (*p* = 0.003 and *p* = 0.004, respectively). These results revealed that CFB between followers does not impair the synchronization accuracy between the leader and followers during synchronization to the human leader.

We did not find an effect of the CFB between the followers on the SDs of the normalized SEs. However, we found that the SDs were larger under the human leader conditions than under the metronome conditions, and the SDs were even larger under the HF conditions than under the HNF conditions (Fig. 2d). A three-way mixed-design ANOVA with pitch, leader type, and between-follower FB showed no significant difference for between-follower FB (*p* = 0.065) but showed a difference for leader type (*p* < 0.001). Multiple comparisons revealed significantly larger SDs of SEs under the HNF and HF conditions than under the M conditions (*p* = 0.001 and *p* < 0.001, respectively) and even larger SDs of SEs under the HNF conditions than under the HNF conditions (*p* < 0.001). Thus, the variability of SEs in synchronization with a human leader was larger than that with the isochronous tones, and the feedback from the followers to the leader enlarged the SE variability.

### Accuracy and variability of leaders’ tapping performance

**Figure 3:**
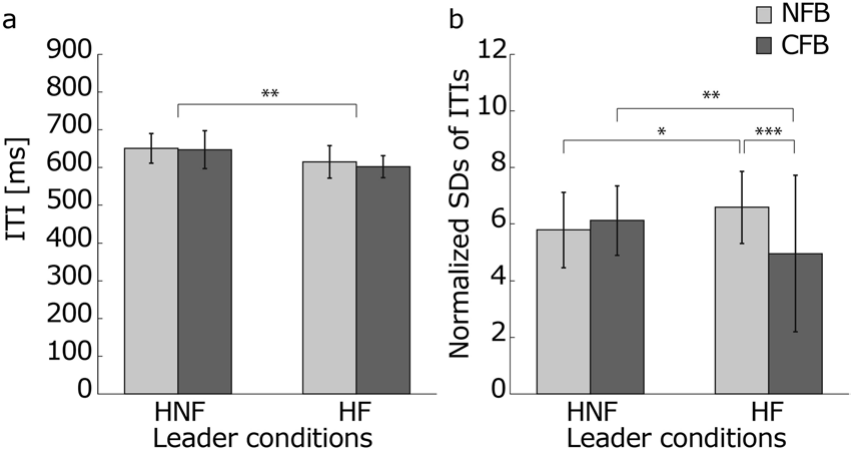
The temporal accuracy and variability of the leader’s tempo. (**a**) Averaged ITIs of the leaders. These values indicate the accuracy of the leader’s tempo keeping. The CFB between the followers did not change the leader’s tempo. The feedback from the followers to leaders increased the speed of the leader’s tempo. (**b**) Means of the coefficient values of the leader’s ITIs. This index depicts the leader’s tempo variability. A value of 0.1 means that the SD of the leader’s ITIs is 10% of the averaged leader’s ITIs in each condition. The CFB between the followers increased the tempo variability of the leader without feedback from the followers but decreased that of the leader with feedback from the followers. The error bars indicate the SDs between trials. *, ** and *** represent *p* < 0.05, *p* < 0.01 and *p* < 0.001, respectively.

Next, we investigated the leaders’ accuracy of tempo keeping and their tempo variability. The CFB between the followers did not affect the means of the leader’s ITIs (Fig. 3a). In addition, the averaged leader ITIs were smaller under the HF conditions than under the HNF conditions. A two-way, repeated-measures ANOVA with the two human leader types and the between-follower FB showed no significant difference in the CFB between followers (*p* = 0.223). There was a significant difference in leader type (*p* = 0.003). Thus, the CFB between followers did not increase the leader’s tempo, but the feedback from the followers to the leader did. It is well known that the tempo gradually increases or decreases in a continuous tapping task in a range of tempos^25,30,37^. For the present study, the leader performed this task under the HNF condition, and the leader’s tempos increased. In addition, the feedback from the followers to the leader increased the leader’s tempo. With the acceleration of the leader’s tempo, the followers’ tempo also increased (Fig. 2a).

The SDs of the leader’s ITIs were not changed by the CFB between followers under the HNF conditions but decreased under the HF conditions (Fig. 3b). In addition, the SDs of the leader’s ITIs increased because of the feedback from the followers to the leader under the NFB conditions but decreased under the CFB conditions. We performed a two-way repeated-measures ANOVA for the SDs of the leader’s normalized ITIs. There was a significant interaction between leader type and between-follower FB (*p* < 0.001). We found that under the NFB condition, the normalized SDs of the leader’s ITIs were significantly larger under the HF condition than under the HNF condition (*p* = 0.030), and under the CFB condition, the normalized SDs of the leader’s ITIs were significantly smaller under the HF condition than under the HNF condition (*p* = 0.005). Moreover, under the HF condition, the normalized SDs of the leader’s ITIs were significantly smaller under the CFB condition than under the NFB condition (*p* < 0.001). These results revealed that the feedback from the followers to the leader increased the SDs of the leader’s ITIs under the NFB conditions but decreased under the CFB conditions. That is, when the followers separately synchronized with the leader, the feedback from the followers destabilized the leader’s tempo. In contrast, when the followers synchronized to the leader together with the partner, the CFB stabilized the leader’s tempo.

### Temporal dependencies of tempos

Third, to investigate the temporal dependencies of the tempos between the leader and the followers and between followers, we used windowed cross-correlation^38^, which revealed temporally local dependence between two time series. If the correlation between the ITIs of two participants was positive at lag −1 or lag 1, one participant adjusted his or her ITI to the partner’s previous ITI. That is, if the partner’s ITI increased or decreased, the participants increased or decreased their next ITI, respectively. If both correlations at lags −1 and 1 were positive, the participants mutually tracked the partner’s ITI. A positive correlation at lag 0 means that the participants’ ITI changed in the same manner in real time. On the other hand, a negative correlation at lag 0 shows real-time compensation between the participants’ ITIs. When one participant increased his/her ITI, the other participant decreased it in real time.

#### Dependencies between human leader and followers

**Figure 4:**
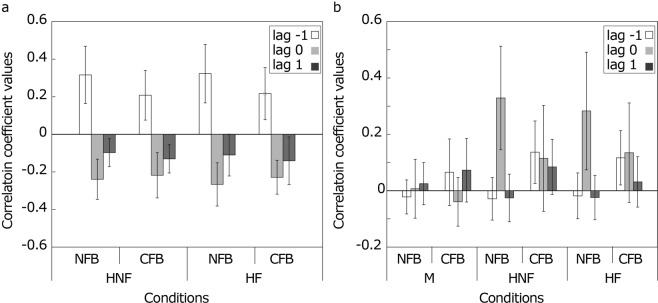
Means of the windowed cross-correlations. (**a**) Tempo dependencies between the human leader and the followers. The positive values at lag −1 in each condition revealed that the followers adjusted their ITIs to the leader’s last ITIs. (**b**) Tempo dependencies between the followers. Under the M-NFB conditions, there was no specific relationship between the followers. Under the M-CFB conditions, a hyper-follower relationship was found, although the correlation values were small. In the HNF and HF conditions, positive correlations at lag 0 were observed under the NFB condition, and positive correlations at all lags were found under the CFB conditions. The error bars indicate the SDs between trials.

**Figure 5:**
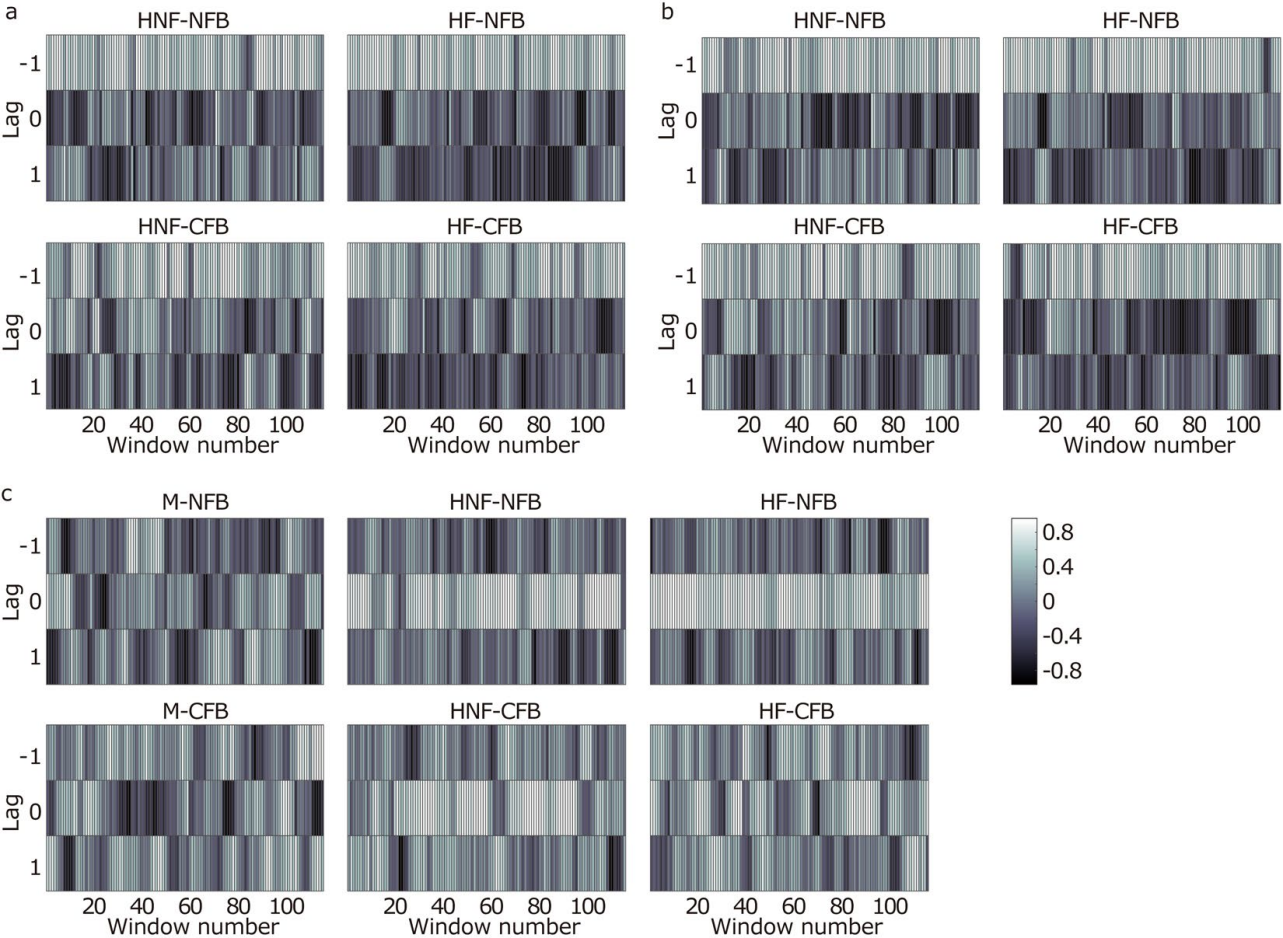
Sample time series of the windowed cross-correlations in the same group; (**a**–**c**) show time series of the windowed cross-correlations in one trial between the human leader and follower 1, between the human leader and follower 2, and between followers 1 and 2, respectively. These graphs show the transitions of the temporal local dependencies between the participants. In a and b, positive correlations at lag −1 were found through the trials in all conditions. In c, there were positive correlations at lag 0 through the trials under the HNF and HF conditions. In addition, more positive correlations at lags 1 and −1 were found under the CFB condition than under the NFB condition in each leader type, although positive correlations were not always observed throughout the trials.

The windowed cross-correlations between the human leader and the followers at lag −1 showed positive values (Fig. 4a). The positive correlation at lag −1 between the leader and the followers means that the followers’ ITIs tracked the leader’s previous ITIs, which is also observed in the synchronization with a sequence including unpredictable perturbation variability^30,39–41^. These positive correlations were observed through the trials in all human conditions (Fig. 5a,b). To compare the correlations between the conditions, we conducted a three-way mixed-design ANOVA for each lag. Only the main effects of between-follower FB were significant in all lags (*p* < 0.001 for lag −1, *p* = 0.011 for lag 0, and *p* = 0.003 for lag 1). Thus, the leader-follower-type relation between the leader and the followers was well constructed in synchronization with the human leader. In addition, the CFB between the followers weakened the correlations of ITIs between the leader and the followers. This is because the effect of the partner’s timing on the follower’s tempo reduced the effect of the leader’s timing even though the followers tended to ignore their partner’s responses.

#### Dependencies between followers

Under the M-CFB condition, the correlation coefficient values at lags 1 and −1 were positive. Under the HNF-NFB and HF-NFB conditions, positive correlations were observed at lag 0. In addition, under the HNF-CFB and HF-CFB conditions, positive correlations were shown at all lags. The tempo dependencies between the followers are shown in Fig. 4b. Under the M-NFB condition, the correlation coefficient values for all the lags were close to zero. This result is not surprising because there were no direct or indirect relationships between the followers under this condition. For each lag, we conducted a two-way repeated-measures ANOVA with leader type and between-follower FB. For lags −1 and 1, there were significant differences in between-follower FB (*p* < 0.001 and *p* = 0.045, respectively). Thus, at lags −1 and 1, the CFB between the followers shifted the correlations at lags −1 and 1 to the positive direction. Thus, the followers under the CFB condition mutually tracked their ITIs, although the followers had been asked to ignore their partner in our experiment. However, the correlations were low, and this positive correlation was not always observed throughout the trials (Fig. 5c), unlike a previous study^18^ in which the participants were asked to keep a constant tempo with a partner. In the present study, the followers’ task was to synchronize to the human leader. Therefore, the relationship between the followers became weak compared to the previous study. For the correlation at lag 0, there was a significant interaction between the leader type and the between-follower feedback (*p* = 0.003). First, we found a significant simple effect of leader type under the NFB conditions (*p* = 0.016). Multiple comparisons showed that under the NFB conditions, the correlation coefficient values under the HNF and HF conditions were higher than those under the M conditions (*p* = 0.021 and *p* = 0.037, respectively). In addition, the lag-0 coefficient values under the NFB conditions were higher than those under the CFB conditions at the HNF and HF conditions (*p* < 0.001 and *p* = 0.005, respectively). These results revealed that the correlations at lag 0 under the NFB conditions in the human leader conditions were higher than those under the other conditions. These positive correlations were observed because the followers’ ITIs adapted well to the human leader’s last ITI simultaneously (Fig. 4a). Under the M-CFB condition, the average correlation at lag 0 was negative. Thus, the followers tended to compensate for their ITIs in real time. However, the correlation coefficient value was smaller in our study than in a previous study^18^. This is because of the task difference, as mentioned above. In contrast, the average correlations at lag 0 were positive under the HNF-CFB and HF-CFB conditions, although we found no significant differences between leader type under the CFB conditions (*p* = 0.114). Under the HNF-CFB and HF-CFB conditions, the follower’s relationship with the leader and the partner was integrated. That is, the strong positive correlation by tracking to the leader’s tempo, which was observed under the HNF-NFB and HF-NFB conditions, and the weak negative correlation by the real-time compensation of ITIs between the followers were mixed. Therefore, the differences between the leader types were not found under the CFB conditions.

### Follower’s phase correction responses to leader and partner

**Figure 6:**
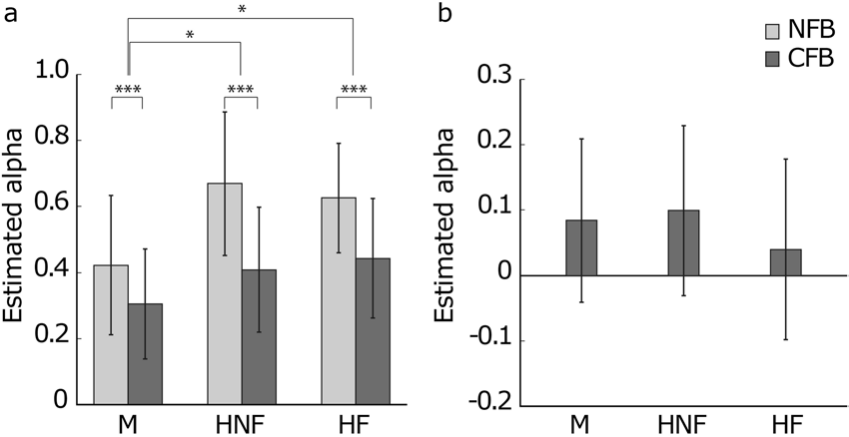
(**a**) Estimated alpha of the followers’ PCRs to the leader. Alpha means the constant for the PCR, and a large alpha shows a strong PCR. Under both human leader conditions, the PCRs were higher than under the metronome conditions. In all leader types, the CFB between the followers decreased the PCRs. (**b**) Estimated alpha of the followers’ PCRs to the partner under CFB conditions. There were no significant differences between the conditions. The error bars indicate their SDs between trials. * and *** represent *p* < 0.05 and *p* < 0.001, respectively.

Finally, to investigate the temporal dependencies of the participant’s timing correction, we estimated the degree of phase correction response (PCR) (Fig. 6), which is timing correction using asynchrony between external signals and one’s own taps. We performed this estimation, using Jacoby’s estimation method^42,43^, which estimates the degree of PCRs with or without period corrections. Two independent error corrections in the rhythm synchronization with the external sequences have been observed: phase correction and period correction (see reviews^1,2^). The period correction is tempo change occurring when the tempo of the external sequences is changed. Thus, we used the model including only phase correction to estimate PCRs from the followers to the metronome and the partner under the M condition because the followers’ tempo change was not observed in this condition (Fig. 2a). We estimated the PCRs from the followers to the leader and the partner using the model including both phase correction and period correction because the leader’s and follower’s tempos changed under the HNF and HF conditions (Fig. 2a).

The PCRs were smaller under the M conditions than under the HNF and HF conditions (both *p* = 0.029) (Fig. 6a). In addition, the CFB between the followers decreased the PCRs under all leader conditions (*p* < 0.001). Thus, the follower’s PCRs to the leader was the smallest in the M-CFB condition. Furthermore, the follower’s PCRs to the partner under the CFB condition were not significantly different between the human leader conditions (*p* = 0.157) (Fig. 6b).

## Discussion

This study aimed to investigate the contribution of the CFB between the followers to performance accuracy and stability in synchronization with a human leader. In addition, we investigated the temporal dependencies between the leader and followers and between followers, which affected accuracy and stability. The CFB between the followers did not impair the tempo-keeping accuracy of the leader or the followers or value or variabilities of the SEs. Moreover, the CFB between the followers improved the tempo stability of the followers and the leader with the followers’ feedback. The windowed cross-correlations between followers’ ITIs showed followers’ tempo tracking to the leader and mutual tempo compensation between followers. In addition, the CFB between the followers weakened the tempo dependencies from the followers to the leader. The CFB weakened the PCR from the followers to the leader, although the PCRs between the followers were not changed by the CFB.

The improvement in the leader’s and the followers’ tempo stability from the CFB between the followers under the human leader conditions (Figs 2b and 3b) was caused by the mutual tempo tracking between the followers (Fig. 4b). This mutual tracking was observed between players following a leader in a string quartet of professional musicians^14^. Our result suggests that the mutual tempo tracking also contributed to the stability of the tempo in the ensemble performance. In addition to the mutual tempo tracking between followers, the followers’ tracking of the leader contributed to tempo stability (Fig. 4a). When there were positive correlations at lags −1, 0, and 1 between the followers’ tempos (Fig. 4b), the two followers’ tempos gradually and smoothly changed in the same fashion. This tempo coupling between followers decreased the variability of their own and the leader’s tempos. However, the stronger feedback from the followers to the leader under the NFB conditions than under the CFB conditions (Fig. 4a) impaired the tempo stability of the leader. The tracking was stronger when the followers separately tracked the leader than when the followers interacted, and then, the leader’s tempo variability increased because of the feedback from the followers (Fig. 3b). Thus, the stronger feedback from the followers to the leader under the NFB conditions than under the CFB conditions worked as a distractor for the leader. The interaction between followers weakened this strong tracking and prevented the leader’s tempo variability from increasing. These results suggest that for tempo stability in temporal coordination such as an ensemble, the balance of the coupling strength with the leader and the partner is important.

Under all conditions, the means of the normalized SEs were negative (Fig. 2c). People usually show NMAs during synchronization with a metronome using finger tapping^1,2,10,35,36^. The results in our experiment show that NMAs were also observed during synchronization with human leaders, regardless of whether a partner was present. Under the M condition, we found an increase in the NMA between the leader and the followers by the CFB between the followers, as in a previous study^3^. This was because the followers’ finger tap timing was influenced by the partner’s feedback, which came before the metronome tones, on average. When distractor tones were presented with target tones that participants were asked to synchronize with isochronous tones, their tap timing shifted in the direction of the distractors^5–7^. The partner’s responses that occurred before the metronome tone worked as distractor tones under the M-CFB condition. However, in synchronization with the human leader, the synchronization accuracy of the followers to the leader was not disturbed regardless of the presence or absence of the CFB between followers. The followers tended to synchronize their taps to the human leader’s taps more strongly than to the metronome tones; thus, the effect of the partner’s feedback on the synchronization error decreased in the human leader conditions. The PCR results corresponded to this hypothesis (Fig. 6a,b). The intensity of the follower’s PCR to the metronome was weaker under the M-CFB condition than under the other conditions. This relatively small PCR to the leader caused the increase in NMA under the M-CFB condition. On the other hand, the larger PCRs to the leader in the human leader conditions than in the metronome conditions prevented an increase in the NMA by the CFB between the followers in the human leader conditions. The followers’ intention to follow the leader increased the PCR from the followers to the leaders under the human leader conditions. Compared to an isochronous sequence, the PCR was weaker in synchronization with a metronome containing a timing shift and with an “adaptively timed” sequence whose timing was changed along with the participant’s timing^44^. Therefore, the strong PCR in synchronization with the human leader compared to the metronome was caused not by the irregularity in the leader’s tap timing but by the followers’ intention to synchronize with the leader’s timing. The strong PCR by the followers’ intention to follow the human leader prevented an NMA increase. In addition to the followers’ intention to synchronize with the leader, their intention to ignore the partner’s timing contributed to preventing an NMA increase. The CFB between followers decreased the PCRs from the followers to the leader in all leader conditions. However, the PCRs under the human leader conditions were significantly large to prevent an NMA increase. If the followers did not intend to ignore the partner, the PCR from the followers to the human leader would decrease because of the CFB more than our results revealed, and then, the NMA would increase. In the context of a musical ensemble, this intention to follow the leader and the other player would contribute to the synchronization accuracy. Note that even if the followers tried to ignore their partner, they could not eliminate their partner’s effect on their rhythm production (Figs 4b and 6b). The tap timing in the synchronization with a metronome was affected even by the unconscious timing perturbation of the metronome^5–7^. Thus, players in a musical ensemble could not completely eliminate the effect of their partner’s sound, and the weaker relation between the followers compared to that between the leader and the followers would remain even if they ignored the partner, which is needed for the smooth rhythm change and the prevention of an NMA increase as mentioned above.

**Figure 7:**
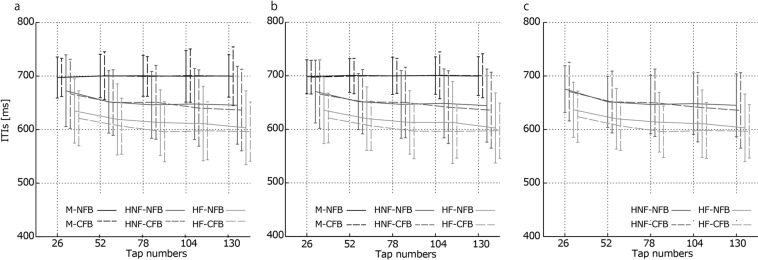
Average ITIs for every 26 taps of (**a**) follower 1, (**b**) follower 2, and (**c**) the leader. These graphs show the average tempo transitions in the trials. In the HNF conditions, the tempos of the followers and the leader gradually increased throughout the trials. In the HF conditions, the tempos of the followers and the leader increased sharply at the beginning of the trials and then increased gradually. Under the HF-CFB condition, the accelerations of the followers’ and leader’s tempos seemed to stop. The error bars indicate their SDs between trials.

Although the CFB between followers did not affect the accuracy of tempo keeping, the feedback from the followers to the leader accelerated both the leader’s and followers’ tempos (Figs 2a and 3a). As mentioned above, the tap timing was shifted in the direction of the distractor timing^30,39,41^. Therefore, when the leader perceived feedback from followers, the leader’s tempo accelerated with the followers’ taps (which tended to occur before the leader’s taps), and the followers’ tempo accelerated according to the leader’s tempo acceleration. Okano *et al*. found that the tempo gradually increased through a trial in an SC task using finger tapping between two people^22^. To investigate whether such a gradual increase in tempo was observed in the present experiment, we calculated the mean ITI transition every 26 taps from the first tap to the 130th tap (Fig. 7). Under the HF-CFB condition, the acceleration seemed to stop in the middle of the trials. Therefore, the human leader could halt the tempo acceleration in the synchronization between people when the tempo increased. The extent to which the human leader keeps the tempo in rhythm synchronization should be investigated in future works. However, it would be difficult to completely prevent acceleration even if there were a leader whose role was to keep the target tempo of the music. In fact, the acceleration in relation to the tendency of tempo to increase, rushing, is often observed in musical ensembles^45^. To prevent rushing, musicians need strong skill in keeping a constant tempo. The ability of tempo keeping was found to be higher for highly trained musicians than for amateurs^9^.

Finally, we did not find any effect of pitch difference between followers’ feedback. When the target and the distractor sequences were presented at the same time, the magnitude of the pitch differences between the two sequences did not affect the PCR^7^. Our results for the pitch difference in the rhythm synchronization between people correspond to previous results for synchronization with a metronome. However, no effects of pitch differences on tap timing were observed in a previous study^7^ or our experiment because relatively high pitch tones, from 500 Hz to over 2,000 Hz, were used. Hove *et al*. investigated the effect of low pitch, which corresponded to the pitch of bass-ranged instruments^46^. They found stronger phase correction to rhythmic auditory stimuli with a low pitch (196.0 Hz) than with a high pitch (466.2 Hz). This result suggests that the sounds by low-pitch instruments could efficiently lead the rhythm in ensembles. In a professional string quartet, the first violin with a high pitch and the cello with a low pitch simultaneously led the second violin and the viola^14^. The cello with a low pitch could easily lead the rhythm because it has a stronger effect on the other players than the first violin with a high pitch does. On the other hand, the appropriate balance of the following players’ intention to follow the leader and the other players, as mentioned above, would be needed for the first violin to lead the rhythm in ensembles.

In conclusion, in synchronization with the human leader, the CFB with the partner who synchronized with the leader contributed to the performance of rhythm production. The CFB between the followers decreased the followers’ and leader’s tempo variability. The improvement in the leader’s and followers’ tempo stability was caused by the followers’ weak mutual tempo tracking via the CFB and the followers’ strong tempo tracking with the leader. In addition, the stronger PCRs from the followers to the human leader than those to the metronome prevented the increase in the NMA by the CFB between the followers, which was shown in the synchronization with the metronome. The results suggest that players in ensembles should appropriately control the balance between influences from the leader and partners. That is, the players should strongly attend to the leader and should direct less attention to the partners. In future work, rhythm synchronization between highly trained musicians should be investigated. The rhythm synchronization performance between musicians is considered to be higher than non-musicians. The high performance of the musicians could be caused by their different balance control of attention to the leader and the partners compared to the non-musicians. The musicians might attend more strongly to the leader and more weakly to the partners than the non-musicians.

## Methods

### Participants

Eighteen people (2 women and 16 men) participated in this experiment and were divided into groups of six. They ranged in age from 21 to 32, and all were right-handed. All participants had no auditory or movement impairment. This experiment was conducted in accordance with the Declaration of Helsinki and authorized by the ethical committee of the University of Tokyo. Written informed consent was obtained from all participants.

### Apparatus and stimuli

The participants’ responses were measured by pressure sensors (PH-464; DKH Corp.). The auditory stimulus was presented via headphones (ATH-T200; audito-techica). All auditory stimuli were generated by a sound generator (custom-made, DKH Corp.). All signals were measured and generated by the interface (custom-made; DKH Corp.) controlled by a special programme (DKH Corp.) on a PC (Dimension 8300; Dell Corp.). All signals were measured at a one-msec resolution. The auditory stimuli for pacemakers were 500 Hz pure tones, and their duration was 100 msec. The metronome’s stimuli and the human leaders’ responses were presented to the followers as 500 Hz pure tones. Those of the two followers were presented to the leader and the other follower as 1,000 and 2,000 Hz pure tones, respectively.

### Task and conditions

The task was to synchronize finger tapping using the right index finger with rhythmic stimuli from a leader with and without feedback from a partner. We used three leader-type conditions: the M, HNF, and HF conditions. In the M condition, two followers tapped their right index fingers to synchronize with a constant-tempo (700 ms) metronome at the same time. In the two human leader conditions, the human leader kept the 700 ms tempo by finger tapping, and the followers’ task was to synchronize with the leader. For the HNF condition, the leaders did not receive any external stimuli. For the HF condition, the leaders were exposed to stimuli related to both followers’ responses. In all leader-type conditions, the followers performed the task with and without the feedback stimuli from the other follower. We call the two between-follower-FB conditions the CFB and NFB conditions. Under the CFB condition, the followers received feedback from their partner. Under the NFB condition, the followers synchronized with the leaders without feedback from their partner.

### Procedures

Three participants were randomly categorized as a leader and two followers, whose responses were presented to the other participants as 500, 1000 and 2000 Hz auditory stimuli, respectively. The participants were seated at each desk and isolated by partitions. The volume of the auditory stimuli via the headphones was adjusted to a comfortable level for each participant and fixed throughout the experiment. The participants closed their eyes and wore an eye mask during the tasks. In addition, they were asked to not move their head or body except for their index finger. Under all conditions, the participants started their finger tapping following the 8 stimuli of the constant-tempo pacemaker (700 ms). After the 8 stimuli, the pacemaker stopped. Under the M conditions, the constant-tempo metronome (700 ms) started after the 8 stimuli of the pacemaker, and the followers were then asked to synchronize with the metronome. Under the HNF and HF conditions, the leaders were asked to keep the pacemaker’s tempo, and the followers tapped to synchronize with the human leaders. Additionally, the leaders were instructed to ignore the auditory stimuli from the followers under the HF conditions. The followers were also asked to ignore the auditory stimuli from the other follower under the CFB conditions.

We prepared blocks for the metronome and human leader conditions. For the M conditions, each between-follower condition (M-NFB and M-CFB) was performed twice in one block. For the human leader conditions, each condition (HNF-NFB, HNF-CFB, HF-NFB, and HF-CFB) was conducted twice in one block. Each block was carried out twice. Therefore, all conditions were performed four times per group. Half of the groups started with the two metronome blocks and then did the two human leader blocks. The order of trials in a block was randomized. The other half was carried out in the inverse order. Thus, the trial orders were counter-balanced between the groups. We conducted practice trials for all conditions. These practice trials were performed twice for one condition. The trial lengths of practice and experiment were 34 seconds and 116 seconds, respectively. The experiment lasted approximately two and half hours per group, including the rest time.

### Statistics

Only the onset tapping times were included in the analyses. The first 10 taps of each tapping sequence were eliminated, and the next 120 taps were used for the analyses. All statistical tests were performed using R version 3.2.4. To evaluate sphericity, the Greenhouse-Geisser method was used. We transformed the coefficient correlation values using Fisher’s z before performing ANOVA. All *post hoc* tests were conducted using the Shaffer Method^47^. The significance level was set at 0.05 for all tests.

The mean and variability of the SEs increased when IOIs of external stimuli increased^30–34^. Therefore, the SEs between the leader and the followers were normalized as follows, where *t*_*L*_(*n*) and ${t}_{{F}_{i}}(n)$ are the onset timing of the leader’s or the follower’s tap:

1 $$IT{I}_{L}(n)={t}_{L}(n+1)-{t}_{L}(n)$$

2 $$S{E}_{L{F}_{i}}(n)={t}_{{F}_{i}}\,(n)-{t}_{L}(n)$$

3 $$normalizedS{E}_{L{F}_{i}}\,(n)=\frac{S{E}_{L{F}_{i}}\,(n)}{IT{I}_{L}(n-1)},$$

where *i* represents the followers (*i* = 1, 2). *ITI*_*L*_(*n*) is the leader’s n-th ITI, and $S{E}_{L{F}_{i}}\,(n)$ is the n-th SE between the leader and the follower. The n-th normalized SE is the n-th SE divided by the (n − 1)-th ITI of the leader.

To investigate the temporal dependency between participants, we computed windowed cross-correlation^38^ of ITIs between the leader and each follower and between the followers. The moving window size was six taps, the maximum lag was three, and the increment of window and lag was one. The windowed cross-correlation shows the temporal local dependency between two time series. For cross-correlation analysis, the *stationarity*^48,49^ of two time series is usually assumed. The stationarity assumption means that the statistical properties, such as the means and the SDs, do not change across the entire period of the time series. However, this assumption is usually not maintained in the time series of rhythm production with other people. The windowed cross-correlation method does not require the stationarity of time series, but it requires the local stationarity of time series and reveals the local dependencies between two time series, which appear over time in the process.

To estimate the PCR constant of the followers to the leader or the partner, we used *bounded generalized least squares* (bGLS), which is a parameter estimation method for various linear sensorimotor synchronization models proposed by Jacoby *et al*.^42,43^. For the two noise components of the whole variability of tapping, timekeeper and motor variance, this method assumed that the timekeeper variance was larger than the motor variance^50^. For the calculation of the followers’ PCRs, we used the code published by the authors^43^. For the followers’ PCRs to the metronome, we used the phase correction model for a single person and multiple people synchronizing with an isochronous sequence. For the followers’ PCRs to the human leader or the partner, we calculated the phase and period correction model for a single person synchronizing with a non-isochronous sequence and ensemble. These calculations were conducted in MATLAB 2018a (The MathWorks, Inc.).

## Supplementary information

Supplementary information

## References

[1] Repp BH (2005). Sensorimotor synchronization: A review of the tapping literature. Psychon. Bull. & Rev..

[2] Repp BH, Su YH (2013). Sensorimotor synchronization: A review of recent research (2006–2012). Psychon. Bull. & Rev..

[3] Mates J, Radil T, Pöppel E (1992). Cooperative tapping: time control under different feedback conditions. Percept. & Psychophys..

[4] Smith KR, Kao H (1971). Social feedback: determination of social learning. The J. Nerv. Mental Dis..

[5] Repp BH (2003). Phase attraction in sensorimotor synchronization with auditory sequences: effects of single and periodic distractors on synchronization accuracy. J. Exp. Psychol. Hum. Percept. Perform..

[6] Repp BH (2004). On the nature of phase attraction in sensorimotor synchronization with interleaved auditory sequences. Hum. Mov. Sci..

[7] Repp BH (2006). Does an auditory distractor sequence affect self-paced tapping?. Acta Psychol..

[8] Repp BH (1999). Control of expressive and metronomic timing in pianists. J. Mot. Behav..

[9] Repp BH (2010). Sensorimotor synchronization and perception of timing: effects of music training and task experience. Hum. Mov. Sci..

[10] Aschersleben G (2002). Temporal control of movements in sensorimotor synchronization. Brain Cogn..

[11] Krause V, Pollok B, Schnitzler A (2010). Perception in action: the impact of sensory information on sensorimotor synchronization in musicians and non-musicians. Acta Psychol..

[12] Goebl W, Palmer C (2009). Synchronization of timing and motion among performing musicians. Music. Percept..

[13] Wing AM, Endo S, Bradbury A, Vorberg D (2014). Optimal feedback correction in string quartet synchronization. J. Royal Soc. Interface.

[14] Timmers, R., Endo, S., Bradbury, A. & Wing, A. M. Synchronization and leadership in string quartet performance: a case study of auditory and visual cues. *Front. Psychol*. **5**, Article 645 (2014). 10.3389/fpsyg.2014.00645 PMC4066619 25002856

[15] Ogata, T., Takenaka, T. & Ueda, K. Role of partner’s feedback information in rhythm production. In Miyazaki, K., Hiraga, Y., Adachi, M., Nakajima, Y. & Minoru, T. (eds) *Proceedings of the 10th international Conference on Music Perception and Cognition (ICMPC10)*, 313–316 (Sapporo, Japan, 2006).

[16] Himberg, T. Measuring co-operability in tapping dyads. In Miyazaki, K., Hiraga, Y., Adachi, M., Nakajima, Y. & Minoru, T. (eds) *Proceedings of the 10th international Conference on Music Perception and Cognition (ICMPC10)*, 460–464 (Sapporo, Japan, 2006).

[17] Takenaka T, Ogata T, Ueda K (2006). Temporal co-creation between self and others with multi-sensory inputs. Adv. Eng. Informatics.

[18] Konvalinka I, Vuust P, Roepstorff A, Frith CD (2010). Follow you, follow me: continuous mutual prediction and adaptation in joint tapping. The Q. J. Exp. Psychol..

[19] Pecenka N, Keller PE (2011). The role of temporal prediction abilities in interpersonal sensorimotor synchronization. Exp. Brain Res..

[20] Nowicki L, Prinz W, Grosjean M, Repp BH, Keller PE (2013). Mutual adaptive timing in interpersonal action coordination. Psycomusicology: Music. Mind, Brain.

[21] Hennig H (2014). Synchronization in human musical rhythms and mutually interacting complex systems. PNAS.

[22] Okano M, Shinya M, Kudo K (2017). Paired synchronous rhythmic finger tapping without an external timing cue shows greater speed increases relative to those for solo tapping. Sci. Reports.

[23] Stevens LT (1986). On the time-sense. Mind.

[24] Collyer CE, Broadbent HA, Church RM (1992). Categorical time production: evidence for discret timing in motor control. Percept. Psychophys..

[25] Madison G (2001). Variability in isochronous tapping: higher order dependencies as a function of intertap interval. J. Exp. Psychol. Hum. Percept. Perform..

[26] Peters M (1989). The relationship between variability of intertap intervals and interval duration. Psychol. Res..

[27] Mates J, Müller U, Radil T, Pöppel E (1994). Temporal integration in sensorimotor synchronization. J. Cogn. Neurosci..

[28] Repp BH (2008). Metrical subdivision results in subjective slowing of the beat. Music. Percept..

[29] Fujii S (2011). Synchronization error of drum kit playing with a metronome at different tempo by professional drummers. Music. Percept..

[30] Michon, J. A. *Timing in temporal tracking*. (Institute for Perception RVO-TNO, Assen, Netherlands, 1967).

[31] Semjen A, Schulze H, Vorberg D (2000). Timing precision in continuation and synchronization tapping. Psychol. Res..

[32] Zendel BR, Ross B, Fujioka T (2011). The effects of stimulus rate and tapping rate on tapping performance. Music. Percept..

[33] Repp BH (2012). Comments on “The effects of stimulus rate and tapping rate on tapping performance” by Zendel, Ross, and Fujioka (2011). Music. Percept..

[34] Lorås H, Sigmundsson H, Talcott JB, Öhberg F, Stensdotter AK (2012). Timing continuous or discontinuous movements across effectors specified by different pacing modalities and intervals. Exp. Brain Res..

[35] Miyake, I. Researches on rhythmic activity. In Studies from the Yale psychological laboratory, vol. 10, 1–48 (Yale University, 1902).

[36] Woodrow H (1932). The effect of rate of sequence upon the accuracy of synchronization. J. Exp. Psychol..

[37] Kolers PA, Brewster JM (1985). Rhythms and responses. J. Exp. Psychol. Hum. Percept. Perform..

[38] Boker SM, Xu M, Rotondo JL, King K (2002). Windowed cross-correlation and peak picking for the analysis of variability in the association between behavioral time series. Psychol. Methods.

[39] Schulze, H. H. The error correction model for the tracking of a random metronome: Statistical properties and an empirical test. In Macar, F., Pouthas, V. & Friedman, W. J. (eds) *Time, action, and cognition: Towards bridging the gap*, NATO ASI Series (Series D: Behavioural and Social Sciences), 275–286 (Springer Netherlands, 1992).

[40] Repp, B. H. The embodiment of musical structure: effects of musical context on sensorimotor synchronization with complex timing patterns. In Prinz, W. & Hommel, B. (eds) *Common mechanisms in perception and action: attention and performance XIX*, 245–265 (Oxford University Press, 2002).

[41] Madison G, Merker B (2004). Human sensorimotor tracking of continuous subliminal deviations from isochrony. Neurosci. Lett..

[42] Jacoby N, Keller EP, Repp BH, Ahissar M, Tishby N (2015). Lower bound on the accuracy of parameter estimation methods for liner sensorimotor synchronization models. Timing & Time Percept..

[43] Jacoby N, Tishby N, Repp BH, Ahissar M, Keller EP (2015). Parameter estimation of liner sensorimotor synchronization models: Phase correction, period correction, and ensemble synchronization. Timing & Time Percept..

[44] Repp BH, Keller PE, Jacoby N (2012). Quantifying phase correction in sensorimotor synchronization: empirical comparison of three paradigms. Acta Psychol..

[45] Colson, J. F. Tempo and ensemble precision. In *Conducting and Rehearsing the Instrumental Music Ensemble: Scenarios, Priorities, Strategies, Essentials, and Repertoire*, chap. 8 (Scarecrow Press, 2012).

[46] Hove MJ, Céline M, Bruce IC, Trainor LJ (2014). Superior time perception for lower musical pitch explains why bass-ranged instruments lay down musical rhythms. PNAS.

[47] Shaffer JP (1986). Modified sequentially rejective multiple test procedures. J. Am. Stat. Assoc..

[48] Shao XS, Chen PX (1987). Normalized auto- and cross-covariance functions for neuronal spike train analysis. Int. J. Neurosci..

[49] Hendry D, Juselius K (2000). Explaining cointegration analysis: Part I. The Energy J..

[50] Wing AM (2002). Voluntary timing and brain function: An information processing approach. Brain Cogn..

