# Supplementary material for: Cross-feedback with Partner Contributes to Performance Accuracy in Finger-tapping Rhythm Synchronization between One Leader and Two Followers
Source: Sci Rep. 2019 May 24;9:7800. doi: 10.1038/s41598-019-43352-x (PMC6534596; doi:10.1038/s41598-019-43352-x)
Supplement: Supplementary file 1 — Supplementary information [file 41598_2019_43352_MOESM1_ESM.pdf]

Supplemental information for

Cross-feedback with Partner Contributes to Performance Accuracy in Finger-tapping Rhythm Synchronization between One Leader and Two Followers

Taiki Ogata, Takahiro Katayama, and Jun Ota

Statistical results

The means of the followers' ITIs

We conducted a three-way mixed-design ANOVA for the averaged ITIs, in which the factors were the pitch of auditory stimuli at which the followers' response were presented (1,000 or 2,000 Hz), the leader type, and between-followers-FB. The results were as follows: pitch: $F(1,10) < 0.01$, $p = 0.996$; leader type: $F(1.32, 13.21) = 56.14$, $p < 0.001$; between-followers-FB: $F(1,10) = 4.04$, $p = 0.072$; pitch × leader type interaction: $F(1.32, 13.21) < 0.01$, $p = 0.999$; pitch × between-followers-FB: $F(1,10) < 0.01$, $p = 0.982$; leader type × between-followers-FB: $F(1.33, 13.28) = 2.66$, $p = 0.120$; three-way interaction: $F(1.33, 13.28) < 0.01$, $p = 0.999$. Multiple comparisons showed significant differences between the M and HNF conditions, $t(10) = 4.63$, $p < 0.001$, between the M and HF conditions, $t(10) = 10.59$, $p < 0.001$, and between the HNF and HF conditions, $t(10) = 7.57$, $p < 0.001$.

The normalized SDs of the followers' ITIs

During the same procedure to establish the mean of the ITIs, we compared the normalized SDs of ITIs under each condition. The results were as follows: pitch: $F(1,10) = 1.57$, $p = 0.239$; leader type: $F(1.54, 15.36) = 32.26$, $p < 0.001$; between-followers-FB: $F(1,10) = 6.31$, $p = 0.031$; pitch × leader type: $F(1.54, 15.36) = 0.54$, $p = 0.549$; pitch × between-followers-FB: $F(1,10) = 1.71$, $p = 0.220$; leader type × between-followers-FB: $F(1.31, 13.14) = 1.80$, $p = 0.205$; three-way interaction: $F(1.31, 13.14) = 0.13$, $p = 0.790$. Multiple comparisons revealed no significant differences between the HNF and NF conditions, $t(10) = 1.58$, $p = 0.145$, except those between the M and HNF conditions, $t(10) = 5.26$, $p < 0.001$, and between the HNF and HF conditions, $t(10) = 7.28$, $p < 0.001$.

The means of the normalized SEs

Comparing the means of the normalized SEs under each condition, we performed a three-way mixed-design ANOVA with the pitch, leader type, and the between-followers-FB as factors. The results were as follows: pitch: $F(1,10) = 0.78$, $p = 0.397$; leader type: $F(1.23, 12.28) = 8.18$, $p = 0.011$; between-followers-FB: $F(1,10) = 7.66$, $p = 0.020$; pitch × leader type interaction: $F(1.23, 12.28) = 2.24$, $p = 0.158$; pitch × between-followers-FB: $F(1,10) = 0.08$, $p = 0.788$; leader type × between-followers-FB: $F(1.79, 17.9) = 5.56$, $p = 0.016$; three-way interaction: $F(1.79, 17.9) = 1.41$, $p = 0.269$. The results of the *post hoc* test for the leader type × between-follower-FB interaction were as follows: leader type under the NFB and CFB conditions: $F(1.21, 12.11) = 2.74$, $p = 0.120$ and $F(1.69, 16.92) = 11.03$, $p = 0.001$, respectively; between-followers-FB under the M, HNF, and HF conditions: $F(1,10) = 19.84$, $p = 0.001$, $F(1,10) = 0.24$, $p = 0.638$, and $F(1,10) = 4.73$, $p = 0.055$, respectively. Multiple comparisons revealed no significant difference between the HNF and HF conditions under the CFB condition, $t(10) = 0.72$, $p = 0.486$, but significant differences between the M and HNF conditions, $t(10) = 4.52$, $p = 0.003$, and between the M and HF conditions, $t(10) = 3.71$, $p = 0.004$, under the CFB condition.

The SDs of the normalized SEs

A three-way mixed-design ANOVA was conducted in the same manner as the means of the normalized SEs. The results were as follows: pitch: $F(1,10) < 0.01$, $p = 0.982$; leader type: $F(1.09, 10.85) = 25.3$, $p < 0.001$; between-followers-FB: $F(1,10) = 4.31$, $p = 0.065$; pitch × leader type interaction: $F(1.09, 10.85) = 2.16$, $p = 0.170$; pitch × between-followers-FB: $F(1,10) = 1.37$, $p = 0.269$; leader type × between-followers-FB: $F(1.22, 12.22) = 0.83$, $p = 0.404$; three-way interaction: $F(1.22, 12.22) = 0.06$, $p = 0.857$. Multiple comparisons revealed significant differences between the M and HNF conditions, $t(10) = 4.58$, $p = 0.001$, and between the M and HF conditions, $t(10) = 5.40$, $p < 0.001$, and the HNF and HF conditions, $t(10) = 4.77$, $p < 0.001$.

The means of the leader's ITIs

We conducted a two-way, repeated-measures ANOVA for the means of the leader's ITIs with two human leader types (HNF and HF) and between-follower-FB. A significant main effect of leader type was found, $F(1,5) = 28.06$, $p = 0.003$. There was no significant main effect of the feedback between followers, $F(1,5) = 1.93$, $p = 0.223$, or no significant interaction, $F(1,5) = 0.91$, $p = 0.385$.

The SDs of the leader's normalized ITIs

We also performed a two-way repeated-measures ANOVA for the SDs of the leader's normalized ITIs. The results were as follows: leader type: $F(1,5) = 0.61$, $p = 0.471$; between-followers-FB: $F(1,5) = 26.66$, $p = 0.004$; interaction: $F(1,5) =$

93.41, $p < 0.001$. For the interaction, the simple effects were as follows: leader type under the NFB condition: $F(1,5) = 9.17$, $p = 0.029$; leader type under the CFB condition: $F(1,5) = 22.76$, $p = 0.005$; between-followers-FB under the HNF condition: $F(1,5) = 2.32$, $p = 0.188$; between-followers-FB under the HF condition: $F(1,5) = 662.52$, $p < 0.001$.

The windowed cross-correlations between the human leader and the followers

We conducted a three-way mixed-design ANOVA for each lag. Only the main effects of between-followers-FB were significant in all lags: $F(1,10) = 89.21$, $p < 0.001$ for lag -1, $F(1,10) = 9.74$, $p = 0.011$ for lag 0, and $F(1,10) = 15.64$, $p = 0.003$ for lag 1. Significant main effects of the pitch were not found, $F(1,10) = 0.05$, $p = 0.834$ for lag -1, $F(1,10) = 0.76$, $p = 0.404$ for lag 0, and $F(1,10) < 0.01$, $p = 0.944$ for lag 1. In addition, no significant main effects of the leader type were found, $F(1,10) = 0.07$, $p = 0.797$ for lag -1, $F(1,10) = 1.55$, $p = 0.242$ for lag 0, and $F(1,10) = 0.11$, $p = 0.749$ for lag 1. Furthermore, none of the interactions were significant: pitch $\times$ leader type, $F(1,10) = 0.40$, $p = 0.543$ for lag -1, $F(1,10) = 0.61$, $p = 0.453$ for lag 0, and $F(1,10) = 0.23$, $p = 0.642$ for lag 1; pitch $\times$ the between-followers-FB, $F(1,10) = 0.20$, $p = 0.666$ for lag -1, $F(1,10) = 0.29$, $p = 0.600$ for lag 0, and $F(1,10) = 1.38$, $p = 0.268$ for lag 1; leader type $\times$ between-followers-FB, $F(1,10) = 0.13$, $p = 0.728$ for lag -1, $F(1,10) = 0.85$, $p = 0.378$ for lag 0, and $F(1,10) = 0.04$, $p = 0.840$ for lag 1; and three-way interactions, $F(1,10) = 0.11$, $p = 0.750$ for lag -1, $F(1,10) = 0.06$, $p = 0.817$ for lag 0, and $F(1,10) = 0.66$, $p = 0.434$ for lag 1.

The windowed cross-correlations between the followers

For each lag, we conducted a two-way repeated-measure ANOVA with the leader type and the between-followers-FB. The results were similar between lag -1 and 1. Only the main effects of the between-followers-FB were significant, $F(1,5) = 142.67$, $p < 0.001$ for lag -1 and $F(1,5) = 7.07$, $p = 0.045$ for lag 1. The ANOVA revealed no significant effects of the leader type: $F(1.41,7.06) = 0.73$, $p = 0.468$ for lag -1 and $F(1.76,8.78) = 2.19$, $p = 0.171$ for lag 1, or no significant interactions between the two factors, $F(1.62,8.08) = 3.07$, $p = 0.108$ for lag -1 and $F(1.81,9.04) = 1.84$, $p = 0.214$ for lag 1. The results for lag 0 were as follows: leader type: $F(1.13,5.67) = 7.32$, $p = 0.035$; between-followers-FB: $F(1,5) = 102,24$, $p < 0.001$; leader type $\times$ between-followers-FB: $F(1.96,9.82) = 11.01$, $p = 0.003$. The results of the *post hoc* test for the leader type $\times$ between-follower-FB interaction were as follows: leader type under the NFB condition: $F(1.1,5.5) = 11.31$, $p = 0.016$; leader type under the CFB condition: $F(1.16,5.8) = 3.41$, $p = 0.114$; between-followers-FB under the M condition: $F(1,5) = 3.35$, $p = 0.127$; between-followers-FB under the HNF condition: $F(1,5) = 131.05$, $p < 0.001$; between-followers-FB under the HF condition: $F(1,5) = 22.29$, $p = 0.005$. Multiple comparisons revealed no significant difference between the HNF and HF conditions under the NFB condition, $t(5) = 1.23$, $p = 0.272$, but significant differences between the M and HNF conditions, $t(5) = 4.43$, $p = 0.021$, and between the M and HF conditions, $t(5) = 2.83$, $p = 0.037$, under the NFB condition.

PCRs of the followers to the leader

We performed a three-way mixed-design ANOVA for the estimated alpha of followers' PCRs to the leader with pitch, leader type, and between-followers-FB. The results were as follows: pitch: $F(1,10) = 0.68$, $p = 0.430$; leader type: $F(1.27,12.68) = 9.22$, $p = 0.007$; between-followers-FB: $F(1,10) = 116.86$, $p < 0.001$; pitch $\times$ leader type interaction: $F(1.27,12.68) = 0.40$, $p = 0.584$; pitch $\times$ between-followers-FB: $F(1,10) = 0.08$, $p = 0.783$; leader type $\times$ between-followers-FB: $F(1.43,14.25) = 4.10$, $p = 0.051$; three-way interaction: $F(1.43,14.25) = 0.73$, $p = 0.455$. Multiple comparisons for the leader type revealed no significant difference between the HNF and HF conditions, $t(10) = 0.53$, $p = 0.606$, but significant differences between the M and HNF conditions, $t(10) = 3.15$, $p = 0.029$, and between the M and HF conditions, $t(10) = 3.19$, $p = 0.029$.

PCRs between the followers

We performed a two-way mixed-design ANOVA for the estimated alpha of PCRs between the followers with pitch and leader type under the CFB condition. The results were as follows: pitch: $F(1,10) = 4.12$, $p = 0.070$; leader type: $F(1.6,16.04) = 2.14$, $p = 0.157$; pitch $\times$ leader type interaction: $F(1.6,16.04) = 3.01$, $p = 0.086$;
